# Preoptic Area Modulation of Arousal in Natural and Drug Induced Unconscious States

**DOI:** 10.3389/fnins.2021.644330

**Published:** 2021-02-12

**Authors:** Sarah L. Reitz, Max B. Kelz

**Affiliations:** ^1^Department of Anesthesiology and Critical Care, Perelman School of Medicine, University of Pennsylvania, Philadelphia, PA, United States; ^2^Mahoney Institute for Neurosciences, University of Pennsylvania, Philadelphia, PA, United States; ^3^Circadian and Sleep Institute, University of Pennsylvania, Philadelphia, PA, United States

**Keywords:** preoptic area, sleep, anesthesia, sedation, hypothalamus

## Abstract

The role of the hypothalamic preoptic area (POA) in arousal state regulation has been studied since Constantin von Economo first recognized its importance in the early twentieth century. Over the intervening decades, the POA has been shown to modulate arousal in both natural (sleep and wake) as well as drug-induced (anesthetic-induced unconsciousness) states. While the POA is well known for its role in sleep promotion, populations of wake-promoting neurons within the region have also been identified. However, the complexity and molecular heterogeneity of the POA has made distinguishing these two populations difficult. Though multiple lines of evidence demonstrate that general anesthetics modulate the activity of the POA, the region’s heterogeneity has also made it challenging to determine whether the same neurons involved in sleep/wake regulation also modulate arousal in response to general anesthetics. While a number of studies show that sleep-promoting POA neurons are activated by various anesthetics, recent work suggests this is not universal to all arousal-regulating POA neurons. Technical innovations are making it increasingly possible to classify and distinguish the molecular identities of neurons involved in sleep/wake regulation as well as anesthetic-induced unconsciousness. Here, we review the current understanding of the POA’s role in arousal state regulation of both natural and drug-induced forms of unconsciousness, including its molecular organization and connectivity to other known sleep and wake promoting regions. Further insights into the molecular identities and connectivity of arousal-regulating POA neurons will be critical in fully understanding how this complex region regulates arousal states.

## Introduction

Prior to the twentieth century, sleep was considered to be a passive process, caused not by specific neural circuits but rather by reduced sensory input that led to low levels of brain activity. This thinking shifted in the early twentieth century during a viral pandemic of encephalitis lethargica. In some of the earliest examinations into the neurobiology of sleep and wake regulation, neurologist Constantin von Economo noted lesions in the posterior hypothalamus of his patients with excessive sleepiness. Conversely, others exhibiting lesions in the anterior hypothalamus, suffered from severe insomnia. This led him to propose the existence of a “sleep center” in the anterior hypothalamus and a corresponding “wake center” in the posterior hypothalamus that act in opposition to actively regulate arousal state (von Economo, 1930). Since these original findings, the existence of hypothalamic circuits involved in regulating arousal state has been repeatedly confirmed across a variety of mammalian species.

Since these early investigations, the hypothalamus has been increasingly recognized as a loose confederation of autonomous neurons that regulate many essential social and homeostatic functions (Sternson, 2013; Wu et al., 2014; Scott et al., 2015; Tan et al., 2016; Allen et al., 2017; Leib et al., 2017), including sleep and wake (Szymusiak et al., 2007). More specifically, the preoptic area of the hypothalamus (POA) is known to modulate arousal in both natural (sleep and wake) (Gallopin et al., 2000; Lu et al., 2000, 2002; McGinty and Szymusiak, 2001; Gong et al., 2004; Chung et al., 2017) as well as drug-induced (anesthetic-induced unconsciousness) states (Nelson et al., 2002; Lu et al., 2008; Li et al., 2009; Moore et al., 2012; Liu et al., 2013; Han et al., 2014; McCarren et al., 2014; Zhang Y. et al., 2015; Yatziv et al., 2020). However, the degree to which the same population of neurons within the POA modulates arousal in both sleep and anesthesia is unclear. Failure to properly regulate arousal state can have serious costs, including increased risk of obesity, cardiovascular disease, and impaired cognition from improper sleep/wake regulation (Everson, 1993; Taheri, 2006; Gallicchio and Kalesan, 2009; Vgontzas et al., 2009; Buxton and Marcelli, 2010; Cappuccio et al., 2010; Besedovsky et al., 2012)., as well as intraoperative awareness and delayed emergence from anesthesia (Mesa et al., 2000; Sebel et al., 2004; Mashour and Avidan, 2015; Sanders et al., 2017). Given these consequences of improper arousal state regulation—both in natural sleep/wake and in response to general anesthesia—untangling the circuits by which the brain coordinates arousal state is critical.

In this review, we summarize the current understanding of the POA’s involvement in regulating arousal states, both in natural sleep and wake, and under anesthesia, including a growing body of literature that suggests the POA is not strictly a somnogenic node. We also review the shared circuitry hypothesis of anesthesia, and examine the evidence for and against a shared population of sleep- and anesthesia-modulating neurons in the POA. Further, we discuss the obstacles facing investigations into arousal state regulation by the POA, focusing on the functional and molecular heterogeneity of the region. New technical innovations are also highlighted that should enable more refined targeting of POA neuronal subtypes and greatly enhance our understanding of how this complex region regulates arousal states.

## POA Regulation of Unconsciousness Accompanying Natural Sleep

Regulating the timing and stability of the states of consciousness and unconsciousness is critical for an individual’s health and survival. A complete and extended period of sleep deprivation can result in death, while both total and partial sleep deprivation cause neurobehavioral deficits including diminished cognitive performance, increased risk of obesity and cardiovascular disease, and impaired immune system function, among other effects (Everson, 1993; Taheri, 2006; Vgontzas et al., 2009; Besedovsky et al., 2012; Irwin et al., 2016; Al Khatib et al., 2017; Gaine et al., 2018; Hudson and Van Dongen, 2019; Frau et al., 2020). On the other hand, excessive sleep is also associated with pathology, including obesity, diabetes, heart disease, and increased mortality (Gallicchio and Kalesan, 2009; Buxton and Marcelli, 2010; Cappuccio et al., 2010; Barateau et al., 2017). Thus, regulating arousal to ensure the proper timing and amount of sleep is crucial for normal physiological function. While essential for survival, sleep confers a period of extreme vulnerability, as the unconscious individual is unaware of its surroundings. Thus, the ability to rapidly transition from sleep to wake is crucial in order to defend against external threats and respond to the surrounding environment. Given the importance of these states, an understanding of how the brain properly regulates arousal state is essential. Although the POA was one of the earliest studied regions in regards to sleep and wake regulation, it remains one of the most difficult to untangle.

### POA Involvement in Sleep

The POA can be divided into four anatomically defined regions: the median preoptic area (MnPO), medial preoptic area (MPO), lateral preoptic area (LPO), and ventrolateral preoptic area (VLPO). Early investigations into the role of the POA in arousal state regulation found that broad activation of the POA results in the rapid onset of sleep (Sterman and Clemente, 1962), while lesions of the area significantly decrease sleep (Nauta, 1946; McGinty and Sterman, 1968; John and Kumar, 1998; Lu et al., 2000). Subsequent recordings from individual neurons revealed that, while sleep-active neurons are scattered across the POA, higher densities of these neurons exist in the VLPO and MnPO (Sherin et al., 1996, 1998; Szymusiak et al., 1998; Takahashi et al., 2009). For this reason, the majority of studies examining POA regulation of arousal state has focused on these two subregions, though a small number of more recent studies have investigated the wider POA as well, which will also be discussed. Although GABAergic neurons in the MnPO show increases in activity just prior to the onset of sleep (Suntsova et al., 2007), suggesting a role in sleep initiation, their activity has been shown to be more strongly correlated with sleep pressure, rather than sleep *per se*. Thus, this region will be discussed further in a later section of this review.

#### VLPO

The VLPO contains a small cluster of largely GABAergic neurons that are most active during NREM and REM sleep (Sherin et al., 1996; Szymusiak et al., 1998; Gong et al., 2000, 2004; Alam et al., 2014). These GABAergic VLPO neurons also express galanin, an inhibitory neuropeptide (Sherin et al., 1998). VLPO activity correlates with sleep amount, with the average number of c-Fos-expressing VLPO neurons increasing with more time spent asleep (Sherin et al., 1996). In addition to being sleep-active, VLPO neurons are also sleep-promoting. Chemogenetic and optogenetic activation of galaninergic VLPO neurons significantly increases NREM sleep (Kroeger et al., 2018). While the VLPO is typically associated with NREM sleep, a cluster of GABAergic/galaninergic neurons in the extended VLPO is active during REM sleep and reduces REM when lesioned (Lu et al., 2002).

**FIGURE 1:**
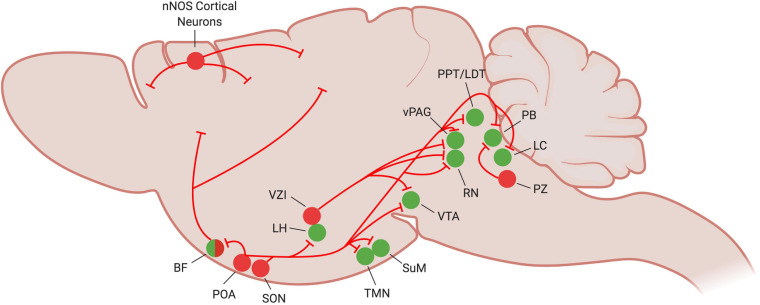
Current understanding of NREM sleep circuitry in the mouse brain. At the onset of NREM sleep, sleep-promoting neurons (red circles) become active and inhibit many of the wake-promoting nuclei of the brain (green circles) to reinforce and stabilize the sleep state. Figure created with BioRender.com.

**FIGURE 2:**
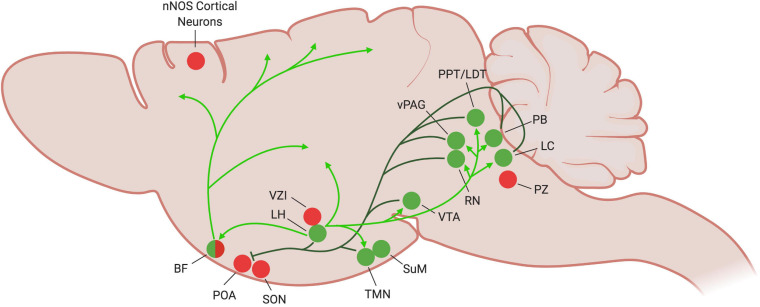
Current understanding of wake-promoting circuitry in the mouse brain. During periods of wake, the wake-promoting nuclei (green circles) become active and inhibit sleep-promoting regions (dark green lines), particularly the POA. Inhibitory projections to other sleep-promoting regions have yet to be established. The orexinergic LH stabilizes the wake state by exciting many of the wake-promoting areas (light green arrows). Many of the wake-promoting regions (LH, TMN, SuM, RN, vPAG, LC) also promote wakefulness by activating the cortex, as illustrated by the BF (light green arrows). Figure created with BioRender.com.

Investigations into the VLPO’s connectivity support the flip-flop switch theory of sleep regulation. The VLPO projects to many members of the arousal-promoting circuitry, including the cholinergic basal forebrain (BF), the lateral hypothalamus (LH), tuberomammillary nucleus (TMN), raphe nuclei (RN), parabrachial nucleus (PB), and locus coeruleus (LC) (Figure 1). It is in turn reciprocally innervated by these same regions (Figure 2; Sherin et al., 1998; Steininger et al., 2001; Chou et al., 2002; Yoshida et al., 2006). VLPO neurons with projections to the LH, RN and ventral periaqueductal gray (vPAG) express c-Fos during sleep (Uschakov et al., 2006, 2009; Hsieh et al., 2011). GABAergic VLPO neurons are directly inhibited by acetylcholine, norepinephrine, and serotonin (Gallopin et al., 2000), providing a mechanism by which the release of wake-promoting neurotransmitters can inhibit sleep-active VLPO neurons to reinforce the waking state. Conversely, the combination of increasing GABA levels in the LC and RN during sleep (Nitz and Siegel, 1997a, b) and enhanced inhibitory galaninergic signaling in the TMN and LC (Schönrock et al., 1991; Pieribone et al., 1995) reduce activity of wake-promoting neurons and further stabilize states of sleep. Furthermore, activation of the VLPO in *ex vivo* brain slices produces GABA-mediated inhibitory postsynaptic potentials in histaminergic TMN neurons (Yang and Hatton, 1997), supporting a role for GABAergic/galaninergic VLPO neurons in promoting sleep. Thus, not only are the VLPO and many wake-promoting regions reciprocally connected, they also mutually inhibit each other, providing support for the flip-flop switch theory of arousal regulation.

#### Wider POA

Lesioning neurons within the POA, including those within the MPO, MnPO, LPO, and VLPO all have been shown to cause insomnia (Szymusiak and McGinty, 1986b; John and Kumar, 1998; Lu et al., 2000; Srividya et al., 2006; Lortkipanidze et al., 2009). This insomnia has been partially reversed by transplantation of fetal preoptic neurons into the lesioned MPO preoptic area (John et al., 1998). Single cell recordings of 128 LPO neurons show that ∼38% are wake/REM-active, ∼43% are sleep-active, and ∼19% are state-indifferent (Alam et al., 2014). Given this evidence that arousal state regulating neurons exist throughout the POA, not just in the VLPO and MnPO, more recent studies have begun investigating the wider POA, including the LPO. A majority of recent studies have focused on the GABAergic/galaninergic population in the region. These populations project to many of the same wake-promoting centers as VLPO and MnPO, including the LH and TMN (Saito et al., 2013; Chung et al., 2017). Activation of GABAergic POA projections to the LH directly inhibits orexinergic neurons in the area (Saito et al., 2013). Furthermore, optogenetic activation of GABAergic POA projections to the TMN promotes sleep, while inhibition promotes wake (Chung et al., 2017). From this population of TMN-projecting, GABAergic POA neurons, they identified 3 subpopulations labeled by neuropeptide markers (cholecystokinin, corticotropin-releasing hormone, and tachykinin 1) that, when optogenetically activated, promote sleep (Chung et al., 2017). Within the LPO, activation of galaninergic neurons promotes NREM sleep, while ablation of this population fragments NREM sleep during the active phase, increasing the number of transitions between the wake and NREM sleep states (Ma et al., 2019). This suggests that galaninergic LPO neurons are essential for consolidated sleep, and are sufficient, but not necessary, for NREM sleep.

### POA Involvement in Wake

**FIGURE 3:**
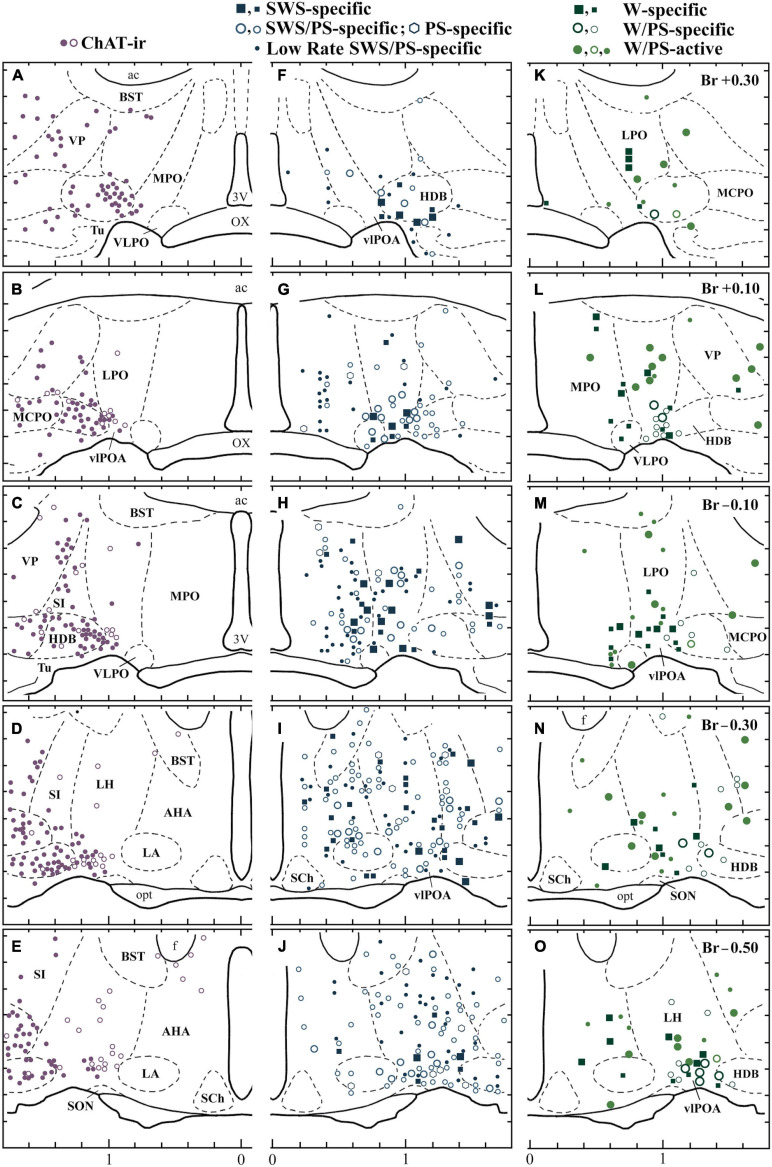
Distribution of sleep-active and wake-active neurons in the POA. Camera lucida drawings of frontal sections (five different planes at 0.2 mm intervals rostral to caudal). **(A–E)** Distribution of ChAT-immunoreactive neurons (dots and circles). **(F–J)** The four groups of sleep-active neurons (squares, circles, circles with a central dot, and dots). **(K–O)** Distribution of waking-specific (squares), waking/PS-specific (circles), and waking/PS-active (dots and thick-lined circles) neurons. The dots and circles in **(A–E)** indicate heavily and faintly stained ChAT-immunoreactive neurons, respectively. The large and small symbols indicate rapidly firing (fast) and slowly firing (slow) neurons, respectively. The thick-lined circles in **(K,M,O)** indicate W/PS-active neurons discharging in close relation to theta waves. 3V, third ventricle; ac, anterior commissure; BST, bed nucleus of the stria terminalis; f, fornix; LA, lateroanterior hypothalamic nucleus; opt, optic tract; OX, optic chiasma; SCh., suprachiasmatic nucleus; SON, supraoptic nucleus; VP, ventral pallidum. Modified from Takahashi et al. (2009). Reprinted with permission from Elsevier.

While many optogenetic and chemogenetic stimulation and lesion/inhibition studies demonstrate a sleep-promoting role for the POA, a growing body of evidence suggests that the region also plays an important role in promoting wakefulness. Single cell *in vivo* recordings illustrate that the POA is much more heterogeneous than originally thought. In addition to sleep-active neurons, the POA contains wake-active and arousal state-indifferent neurons scattered among the sleep-active population (Figure 3; Kaitin, 1984; Szymusiak and McGinty, 1989; Takahashi et al., 2009).

Furthermore, optogenetic activation of GABAergic POA neuronal cell bodies, or glutamatergic POA projections to TMN neurons promotes wakefulness (Chung et al., 2017). A recent study also demonstrated that chemogenetic activation of glutamatergic neurons in the ventral half of the POA increases time spent awake (Vanini et al., 2020). Additionally, we recently showed that chemogenetic activation of tachykinin 1-expressing POA neurons strongly stabilizes and consolidates the waking state, decreasing the number of transitions between sleep and wake, while greatly increasing the average length of wake bouts (Reitz et al., 2020). With this growing evidence supporting a dual role in sleep and wake, it is clear that more work is needed in order to more accurately characterize and understand the POA’s roles in arousal state regulation.

### Homeostatic and Adaptive Arousal State Regulation by the POA

While sleep and wake cycles are strongly regulated by circadian rhythms, a core feature of sleep is that it is also subject to homeostatic regulation. Total or partial sleep deprivation increases sleep drive, ultimately resulting in a period of recovery sleep that is longer and deeper (characterized by enhanced delta power in the EEG) than sleep under unrestricted conditions. The mechanisms by which the brain senses and responds to this homeostatic sleep pressure are not fully understood, though the evidence discussed below points toward the involvement of the POA.

In addition to increasing activity during sleep, both VLPO and MnPO neurons exhibit higher activity in response to sleep deprivation, prior to recovery sleep, suggesting a role in tracking sleep debt (Alam et al., 2014). It was previously thought that VLPO had no role in sensing or responding to sleep pressure, as c-Fos levels were not increased unless animals experienced recovery sleep following sleep deprivation (Sherin et al., 1996; Gvilia et al., 2006). However, more recent studies found that the VLPO exhibits increased c-Fos expression and higher firing rates during sleep deprivation, prior to recovery sleep, suggesting at least a minor role for VLPO in sleep homeostasis as well (Gong et al., 2004; Alam et al., 2014). These may represent two distinct subpopulations within VLPO: one that promotes sleep in response to sleep pressure, and one that maintains sleep (Gallopin et al., 2005).

Similar to VLPO, the MnPO consists largely of GABAergic neurons that are most active during NREM and REM sleep compared to baseline wake (Gong et al., 2000; McGinty et al., 2004; Alam et al., 2014). These neurons are also sleep-promoting, as chemogenetic activation of the GABAergic MnPO promotes sleep (Vanini et al., 2020). However, MnPO activity appears to correlate with sleep pressure rather than sleep amount (Suntsova et al., 2002). The number of c-Fos positive, GAD-expressing MnPO neurons is highest following sleep deprivation but prior to recovery sleep (Gvilia et al., 2006). Additionally, sleep-active MnPO neurons exhibit increased firing rates as sleep pressure builds during sleep deprivation, ultimately firing twice as frequently after 2 h of sleep deprivation compared to baseline sleep. This firing returns to baseline levels as the animal is allowed to sleep (Alam et al., 2014). Together, this suggests a role for the MnPO in tracking sleep debt and maintaining sleep homeostasis.

The MnPO may contribute to sleep homeostasis via inhibition of numerous wake-promoting regions. Like the VLPO, the MnPO is also reciprocally connected to many members of the arousal-promoting circuitry, including the cholinergic BF, LH, TMN, RN, PB, and LC (Steininger et al., 2001; Yoshida et al., 2006; Uschakov et al., 2007). MnPO neurons with projections to the RN and vPAG exhibit increased expression of c-Fos during sleep (Uschakov et al., 2009; Hsieh et al., 2011). Furthermore, activation of MnPO neurons suppresses activity in the wake-active LH, while inhibition of the MnPO had the opposite effect (Suntsova et al., 2007). Similarly, inhibition of MnPO neurons increases c-Fos in the orexinergic LH neurons and serotonergic RN neurons (Kumar et al., 2008), further suggesting functional inhibition of these two wake centers by the MnPO. In contrast to the MnPO and VLPO, sleep-active neurons in the LPO do not show increased activity in response to sleep deprivation (Alam et al., 2014), suggesting this population is not involved in sleep homeostasis.

One possible mechanism by which the POA senses sleep pressure is via a buildup of sleep-generating small molecules, called somnogens, in the brain (Benington and Craig Heller, 1995; Porkka-Heiskanen et al., 1997; Scammell et al., 2001; Basheer et al., 2004). One of the best studied somnogens is adenosine. Adenosine is a byproduct of metabolism in the brain, and brain levels of this molecule increase across waking and during sleep deprivation, decreasing during recovery sleep (Porkka-Heiskanen et al., 1997, 2000; Basheer et al., 2004; Kalinchuk et al., 2011). Application of adenosine to VLPO neurons in *ex vivo* rat brain slices suppresses spontaneous IPSPs (Chamberlin et al., 2003). Furthermore, administration of adenosine A_2A_ receptor agonists into the POA directly activates VLPO neurons and promotes sleep in rats (Ticho and Radulovacki, 1991; Gallopin et al., 2005; Kumar et al., 2013). Additionally, local administration of A_2A_ receptor antagonists into the VLPO reduces sleep deprivation- and recovery sleep-induced firing of VLPO neurons (Alam et al., 2014). Finally, the aforementioned cycles of adenosine levels in the POA that correspond to sleep and wake are present in rats at post-natal day 30 (P30), but not at P22, suggesting that the development of this homeostatic response to sleep loss coincides with the functional emergence of adenosine signaling in the brain. This study also found that sleep-active MnPO neurons are more responsive to sleep deprivation at P30 compared to P22 (Gvilia et al., 2017).

Another small molecule linked to sleep pressure is prostaglandin D_2_ (PGD_2_). PGD_2_ is generated in the leptomeninges and choroid plexus, and is found circulating in the cerebrospinal fluid, where it fluctuates in parallel with the sleep-wake cycle (Huang et al., 2007). Like adenosine, PGD_2_ levels also increase during sleep deprivation (Ram et al., 1997). Administration of PGD_2_ in the subarachnoid space just anterior to the MnPO and VLPO promotes sleep and increases c-Fos expression in VLPO neurons (Scammell et al., 1998; Hayaishi and Urade, 2002). This effect is likely mediated by adenosine, as infusion of PGD_2_ into the subarachnoid space dose-dependently increases extracellular adenosine (Mizoguchi et al., 2001).

As emphasized earlier, regulating the timing and stability of sleep and wake is critical for health and survival, as life-sustaining activities such as eating, seeking shelter, copulating, and escaping from danger all depend upon proper control of arousal. Multiple lines of evidence suggest the POA is capable of integrating diverse inputs, such as temperature and energy status, to produce the most appropriate arousal state response. For instance, local administration of glucose in the VLPO, simulating the “well-fed” state, activates VLPO neurons and promotes NREM sleep, thus providing a potential link between metabolism/energy status and arousal state regulation in the POA (Varin et al., 2015).

Under more extreme conditions, when faced with resource scarcity, some mammals will adapt by initiating energy-conserving survival strategies, such as hibernation or torpor. Recent work highlights that the MPO and LPO are capable of overriding homeostatic setpoints to coordinate profound reductions in metabolism, body temperature, and caloric need to enhance survival (Hrvatin et al., 2020). Work in hibernating ground squirrels has shown increases in c-Fos expression in MPO neurons during entry into hibernation (Bratincsák et al., 2007). Additionally, microinjections of opioid receptor antagonists into the POA of hibernating ground squirrels increased the squirrels’ body temperature and induced arousal from hibernation, suggesting a role for opioid signaling in the POA in hibernation (Yu and Cai, 1993).

Even under less extreme conditions, alterations in body temperature are known to correlate with arousal state. The onset of sleep coincides with a decline in body temperature, and entry into REM is accompanied by near total inhibition of thermoregulatory responses in many species (Krueger and Takahashi, 1997). The POA is poised to be the link between sleep and thermoregulation, as the POA is known to contain thermosensitive and thermoregulatory neurons (Zhao et al., 2017; Ma et al., 2019), with many of the warm-sensitive POA neurons also exhibiting sleep-active firing, while cold-sensitive POA neurons show increased activity during wake (Alam et al., 1995, 1997). Further supporting this link is evidence that local warming of the broad POA or the GABAergic MPO neurons promotes sleep (Roberts and Robinson, 1969; Harding et al., 2018), while local cooling promotes wakefulness (Sakaguchi et al., 1979; McGinty and Szymusiak, 1990).

Recent studies using activity-dependent tagging and reactivation of neurons further reveal a link between arousal state and body temperature. Reactivation of neuronal nitric oxide synthase (*Nos1)*-expressing MnPO/MPO neurons activated during external warming induces both sleep and hypothermia in mice, while reactivation of warming-tagged GABAergic MPO neurons produces NREM sleep (Harding et al., 2018). Further investigations revealed that reactivation of MPO or LPO neurons that were activated during recovery sleep produces profound drops in body temperature (Zhang Z. et al., 2015). Additionally, chemogenetic activation of galaninergic VLPO neurons reduces core body temperature by 4–6°C (Kroeger et al., 2018). Moreover, activation of those same neurons at warmer temperatures (29 and 36°C) decreases latency to NREM and increases NREM duration compared to activation at 22°C (Kroeger et al., 2018). The extreme drop in body temperature resulting from galaninergic VLPO activation also suggests a role for the VLPO in torpor, another state of unconsciousness accompanied by hypothermia, decreased metabolism, and slow wave EEG activity (Berger, 1984).

## POA Regulation of Drug-Induced Unconsciousness

While sleep is a universal, natural form of unconsciousness, unconsciousness also occurs under general anesthesia. However, despite the use of anesthetics for over 170 years and in over 300 million surgeries annually (Weiser et al., 2016), the precise molecular and neuronal mechanisms underlying their hypnotic actions remain poorly understood.

The molecular mechanisms of anesthetic-induced unconsciousness remain unknown due in part to the transient interactions and promiscuous number of general anesthetic binding partners (Eckenhoff, 2001; Urban, 2002), yet all produce an apparently similar behavioral endpoint. While a variety of ion channels are affected by anesthetics, the net effect of anesthetic binding is the hyperpolarization of resting membrane potentials, enhancement of inhibitory neurotransmission, and inhibition of excitatory neurotransmission (Rudolph and Antkowiak, 2004). With the knowledge that anesthetics act on a diverse range of ion channels yet all enhance inhibition and/or inhibit excitation, more recent research has examined the hypothesis that anesthetics may exert their effects not by acting at identical molecular targets, but rather by differentially affecting neurons in a common neural pathway. However, because these receptors are widely expressed throughout the brain, identifying the exact neural circuits critical for producing a state of anesthesia has been difficult.

### The Shared Circuitry Hypothesis

One target that has emerged as a likely mediator of anesthetic hypnosis is the neural circuitry governing sleep and arousal discussed earlier. Although sleep and anesthesia are undoubtedly two distinct states, they share a number of similar traits (Lydic and Biebuyck, 1994). For example, both NREM sleep and anesthetic hypnosis show EEG patterns that include spindles and slow waves (Murphy et al., 2011). Neuroimaging studies have also shown reduced activity in brain regions involved in arousal (Alkire et al., 2000; Vahle-hinz et al., 2001; Detsch et al., 2002) as well as cortical regions involved in association and integration in both states of unconsciousness (Fiset et al., 1999; Franks, 2008).

In addition to these phenotypic similarities, multiple lines of evidence demonstrate a functional relationship between sleep and anesthetic hypnosis. Sleep deprivation reduces the amount of anesthetic required to enter the hypnotic state (Tung et al., 2002), while administration of barbiturates during the waking phase results in a shorter duration of hypnosis (Einon et al., 1976). Furthermore, administration of select anesthetics for prolonged periods does not incur new sleep debt and may actually relieve preexisting sleep debt (Tung et al., 2004; Nelson et al., 2010; Pal et al., 2011; Pick et al., 2011). These findings have led to what is known as the “shared circuitry hypothesis” of anesthesia, which posits that anesthetics exert their hypnotic effects in part by acting on the neural circuitry that regulates endogenous sleep and wake. More specifically, this theory hypothesizes that anesthetics cause unconsciousness via activation of sleep-promoting populations and/or inhibition of wake-promoting populations, rather than by the wet-blanket theory of non-specific, global disruption of CNS function (Lydic and Biebuyck, 1994; Yatziv et al., 2020).

Although a number of studies have implicated sleep- and wake-regulating brain areas in anesthetic hypnosis, controversy remains as to whether the neural circuits, and more specifically, the same neurons shaping sleep and wakefulness actually do influence the anesthetic state *in vivo*. Past work has demonstrated that the POA, in addition to modulating sleep and wake, is also capable of modulating anesthetic-induced unconsciousness (Nelson et al., 2002; Lu et al., 2008; Li et al., 2009; Moore et al., 2012; Liu et al., 2013; Han et al., 2014; McCarren et al., 2014; Zhang Y. et al., 2015; Yatziv et al., 2020). However, the degree to which the same population of neurons within the POA modulates arousal in both sleep and anesthesia is unclear.

### POA Involvement in General Anesthesia

Because the VLPO and MnPO contain the highest densities of sleep-active neurons, the majority of work investigating the role of the POA in anesthetic hypnosis has focused on these two regions, particularly the VLPO.

#### VLPO

Exposure to hypnotic doses of all anesthetics except for ketamine increases c-Fos expression in VLPO (Nelson et al., 2002; Lu et al., 2008; Li et al., 2009; Moore et al., 2012; Han et al., 2014), positioning this region as a potential common mediator of anesthetic hypnosis. Furthermore, c-Fos expression in VLPO is positively correlated with isoflurane dose, suggesting that isoflurane may dose-dependently activate VLPO neurons (Moore et al., 2012). Activation of VLPO under anesthesia may arise from either disinhibition or from direct excitation. This has not been examined for many anesthetics, though isoflurane is known to directly depolarize putative sleep-active VLPO neurons (Moore et al., 2012). However, not every VLPO neuron is activated by isoflurane. Single cell recordings within VLPO reveal two distinct subpopulations: isoflurane-activated VLPO neurons, and isoflurane-inhibited VLPO neurons (Moore et al., 2012; McCarren et al., 2014). The isoflurane-activated neurons are considered to be putative sleep-active as well since they match the *ex vivo* neurochemical phenotype of low-threshold spiking neurons that are inhibited by norepinephrine (Moore et al., 2012); however, formal *in vivo* proof of this potential convergence was not obtained.

On a functional level, VLPO lesions increase resistance to propofol, significantly increasing the time to loss of righting reflex after administration and decreasing the duration of loss of righting reflex (Zhang Y. et al., 2015). Lesions of VLPO neurons also increase wakefulness and decrease isoflurane sensitivity, though only acutely following the lesion. This decreased sensitivity to isoflurane subsequently transitioned to enhanced sensitivity at later timeponts (Eikermann et al., 2011; Moore et al., 2012). The enhanced sensitivity to isoflurane observed at these later timepoints is thought to be the result of sleep deprivation caused by the VLPO lesions, which increases sensitivity to anesthetics as discussed earlier (Tung et al., 2002). Conversely, inhibition of VLPO neurons, via activation of α2 adrenergic receptors, increases behavioral arousal under isoflurane anesthesia (McCarren et al., 2014). Together, these results support a role of VLPO in regulating arousal under anesthesia. However, these studies did not confirm their VLPO modulations also affected sleep/wake activity. Thus, though evidence supports a role for the VLPO in arousal state regulation under sleep/wake and anesthesia, to what degree these two populations converge remains an open question. Recent work supports the existence of two distinct populations within the VLPO, demonstrating that chemogenetic activation of GABAergic VLPO neurons alters sleep-wake architecture without affecting anesthetic sensitivity or recovery time in the same mice (Eikermann et al., 2011; Vanini et al., 2020).

#### MnPO

Evidence for a role of the MnPO in anesthetic hypnosis is much less clear than VLPO. Exposure to isoflurane increases c-Fos expression in MnPO neurons, however, exposure to halothane, pentobarbital, and chloral hydrate do not (Lu et al., 2008; Han et al., 2014). This activation by isoflurane likely results from either disinhibition or secondary activation via VLPO or another anesthetic-activated region, as isoflurane-induced activation of MnPO does not occur in the presence of tetrodotoxin (Han et al., 2014). Additionally, reactivation of MnPO neurons that were active during dexmedetomidine-induced sedation does not promote sleep (Zhang Z. et al., 2015) and activation of GABAergic MnPO neurons that promote sleep do not alter anesthetic state transitions (Vanini et al., 2020), suggesting that anesthetic-activated MnPO neurons are not the same neurons that promote sleep.

#### Broader POA

POA Tac1 neurons that promote and consolidate wakefulness also enhance resistance to isoflurane and sevoflurane anesthesia (Reitz et al., 2020). This increase in resistance is more pronounced on emergence from the anesthetic state compared to induction. Thus, the potent effects of Tac1 activation work to support the waking state over both endogenous as well as anesthetic-induced impairment of arousal. However, chemogenetic inhibition of this population had no effect on either sleep or anesthetic sensitivity. Whether this is due to a technological limitation, a relative quiescence of Tac1 neurons at baseline, or a true indication that POA Tac1 neurons are sufficient but not necessary for promoting wakefulness remains to be seen. Also unclear is whether POA Tac1 neurons play any endogenous role in circuits regulating natural sleep/wake and those regulating anesthetic-induced unconsciousness, or whether POA Tac1 neurons increase resistance to anesthesia via a neural pathway independent from that utilized by general anesthetics.

### POA Involvement in Sedation

Distinct from the deep unconsciousness achieved by general anesthetics such as propofol or isoflurane, dexmedetomidine produces a state of moderate sedation. This moderate sedation shares many properties with natural sleep, with both states characterized by a loss of consciousness, but intact ability to be aroused by external stimuli.

A recent study examined the relationship between sleep and dexmedetomidine-induced sedation in the LPO. Zhang and colleagues show that the same neurons active during dexmedetomidine-induced sedation promote NREM sleep when subsequently re-activated. Zhang’s clever use of the TetTag system provides causal support for the shared circuitry hypothesis in the LPO. Whether these results are true for general anesthetics such as isoflurane or propofol remain unknown. Additionally, re-activation of these dexmedetomidine-active LPO neurons produces a drop in body temperature, linking the POA to thermoregulation in both natural and drug-induced forms of unconsciousness. Finally, deletion of the *Vgat* gene from LPO neurons increases resistance to dexmedetomidine-induced sedation (Zhang Z. et al., 2015), suggesting that GABAergic LPO neurons specifically are required for dexmedetomidine-induced sedation.

## Challenges in Studying Arousal Regulation by the POA

### Molecular Heterogeneity

**FIGURE 4:**
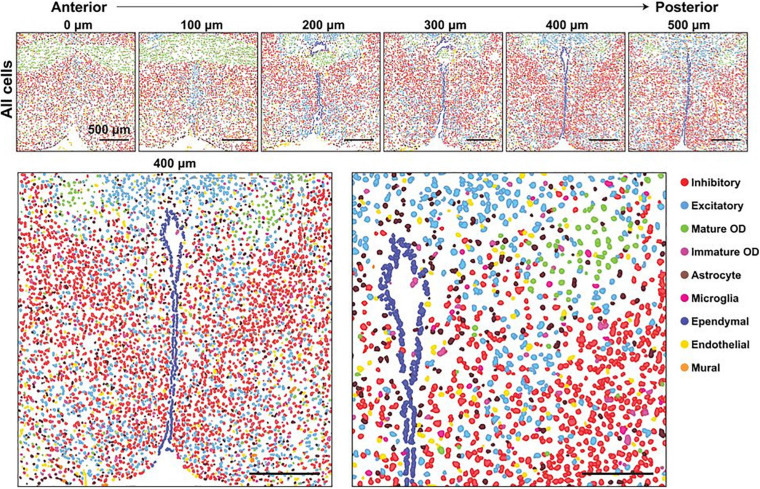
Major cell classes and their spatial organizations in the POA as revealed with MERFISH. **(Top)** Spatial distribution of all major cell classes across sections at different anterior–posterior positions from a single female mouse. Cells are marked with cell segmentation boundaries and colored by cell classes as indicated. Six of the twelve 1.8- by 1.8-mm imaged slices are shown. The 0, 100, 200, 300, 400, and 500 mm labels indicate the distance from the anterior position (Bregma + 0.26). **(Bottom)** Enlarged image of the slice at 400 mm from the anterior position (left) and a further magnified image of the region shown in the gray dashed box (right). Scale bars, 500 mm (left), 250 mm (right). From Moffitt et al. (2018). Reprinted with permission from AAAS.

Perhaps the largest challenge facing the study of arousal regulation by the POA is the immense heterogeneity of the region (Figure 4; Moffitt et al., 2018). In addition to regulating arousal state, the POA is involved in many essential social and homeostatic functions, including thermoregulation, thirst and osmotic homeostasis, parenting, and social play behaviors (Sternson, 2013; Wu et al., 2014; Scott et al., 2015; Tan et al., 2016; Allen et al., 2017; Leib et al., 2017; Zhao et al., 2020). As mentioned earlier, heterogeneity exists even among arousal state regulating neurons in the POA, with sleep-active neurons interspersed among wake-active and arousal state-indifferent neurons (Kaitin, 1984; Szymusiak and McGinty, 1986a; Szymusiak et al., 1998). Recordings in the LPO and MPO found 21% of recorded neurons to be wake/REM-active, 66% to be sleep-active, and 13% to be state-indifferent (Figure 3; Takahashi et al., 2009). Additional recordings of 128 LPO neurons show that roughly 38% are wake/REM-active, 43% are sleep-active, and 19% are state-indifferent (Alam et al., 2014). Similarly, although the VLPO and MnPO are more densely populated by sleep-active neurons, roughly 20% of neurons in each area are estimated to be wake-active (Gaus et al., 2002; Alam et al., 2014).

Because of this heterogeneity, the majority of studies investigating arousal state regulation by the POA have focused on two broad classes of neurons: the inhibitory GABAergic/galaninergic neurons typically shown to be sleep-active, and the excitatory glutamatergic neurons, typically associated with wake. However, the assumption that inhibitory neurons are sleep-active and excitatory neurons are wake-active is not as straightforward as is often assumed. Though the majority of galaninergic VLPO neurons are sleep-active, roughly 20% of the population is actually wake-active (Gaus et al., 2002). Further, of the POA neurons activated during recovery sleep, roughly 15% are glutamatergic (Zhang Z. et al., 2015). These functional differences within the broad class of excitatory POA neurons is further illustrated by work showing that activation of glutamatergic VLPO neurons promotes wake (Vanini et al., 2020), while activation of largely glutamatergic NOS1-expressing MnPO neurons causes entry into NREM sleep (Harding et al., 2018). While activation of inhibitory POA neurons promotes sleep in some studies (Chung et al., 2017; Kroeger et al., 2018; Ma et al., 2019), yet have no effect on sleep or wake in others (Vanini et al., 2020).

As a result, investigations into the role of inhibitory POA neurons in anesthetic-induced unconsciousness have also produced sometimes-opposing results. For instance, isoflurane-induced unconsciousness directly depolarizes and increases expression of c-Fos in putative sleep-active GABAergic neurons within the VLPO (Moore et al., 2012), yet broad activation of this GABAergic population alters sleep-wake architecture (Saito et al., 2013; Kroeger et al., 2018) without affecting the time to anesthetic induction or time required for emergence (Vanini et al., 2020). Together, this emphasizes that these molecular markers traditionally used to distinguish the sleep-active and wake-active populations within the POA are not accurate enough, and that a more refined targeting of POA cell types is needed when investigating arousal state regulation.

The immense heterogeneity of these two neuronal subtypes may underlie these differing results. Recent studies have shown that the molecular marker chosen to access the inhibitory population is one important consideration. The GABA transporter, VGAT, is rarely expressed alongside Vglut2, the glutamatergic transporter. However, GABA synthesis genes *gad1* and *gad2* are sometimes expressed in the same neurons as Vglut2, representing a population of POA neurons capable of coreleasing GABA and glutamate (Romanov et al., 2016; Moffitt et al., 2018). Thus, studies using Gad as a marker of inhibitory neurons in the POA may unintentionally also modulate glutamatergic signaling to an unknown degree. Furthermore, recent single-cell RNA-sequencing of GABAergic and glutamatergic neurons within the POA has revealed an enormous level of molecular diversity within these two groups, consisting of nearly 70 subpopulations clustered based on gene expression (Moffitt et al., 2018). Even more selective neuropeptide markers may be inadequate to conclusively distinguish sleep-from wake-promoting neuronal populations within the POA, as activation of POA Tac1 neurons has been shown to increase sleep in one investigation (Chung et al., 2017), yet strongly enhance and stabilize wakefulness in another (Reitz et al., 2020).

Together, this suggests that a single molecular marker may not be sufficient to accurately distinguish sleep and wake population within the POA. Molecular markers combined with projection-specific labeling may be one method to more accurately identify these populations, as past work has shown that optogenetic activation of GABAergic POA neuronal cell bodies or glutamatergic POA projections to the TMN produces wake, while activation of GABAergic POA terminals in the TMN produces sleep (Chung et al., 2017). However, optogenetic stimulation of GABAergic POA projections to either the habenula or the dorsomedial hypothalamus have no effect on arousal state (Chung et al., 2017), further emphasizing the importance of examination specific axonal targets.

### Methodological Limitations and Considerations

Another set of challenges when studying arousal state regulation by the POA arise from methodological limitations, both from an experimental design standpoint and inherent limitations with the methods themselves. Studies investigating the shared circuitry hypothesis in the POA often use GABA or galanin as markers of the sleep-active neuronal population, yet many fail to confirm that these same anesthetic-activated neurons are also involved in arousal regulation. Thus, though multiple lines of evidence demonstrate that general anesthetics and sedatives modulate the activity of the POA, and even the GABAergic/galaninergic POA, it is still unclear whether the same population of neurons involved in sleep/wake regulation also modulate arousal in response to general anesthetics, or whether these are two separate populations that exist in the same region. Given the uncertainty of the exact role of GABAergic and glutamatergic POA neurons in sleep and wake, it is clear that modulations of sleep and anesthesia must be examined in the same cohort without assuming that neurons expressing a particular molecular marker represent an arousal state-regulating population.

Another important consideration when studying arousal state regulation in the POA is the method of activation used. We recently demonstrated that chemogenetic activation of POA Tac1 neurons using designer receptors exclusively activated by designer drugs (DREADDs) strongly stabilizes the wake state, decreasing the number of transitions between sleep and wake while greatly increasing the average length of wake bouts (Reitz et al., 2020). This is in contrast to previously published results describing NREM-promoting effects when POA Tac1 neurons are optogenetically activated (Chung et al., 2017). It is possible that fundamental differences between the neuronal activation achieved by DREADDs compared to that achieved by optogenetics underlie these contrasting findings. While we will briefly discuss important considerations to take into account when utilizing either method, more detailed comparisons of these techniques can be found in a number of reviews specifically discussing this topic (Aston-Jones and Deisseroth, 2013; Krook-Magnuson and Soltesz, 2015; Vlasov et al., 2018).

When utilizing optogenetic activation, the stimulation frequency is a critical concern. This has been highlighted in recent work demonstrating optogenetic activation of GABAergic POA neurons promotes wakefulness when stimulated at 10 Hz (Chung et al., 2017). However, chemogenetic activation or optogenetic activation at lower frequencies (0.5–4 Hz), which more closely match the endogenous firing rate during NREM sleep, have been shown to promote NREM sleep (Kroeger et al., 2018). This discrepancy is likely due to a conduction block resulting from stimulation above 8 Hz, functionally inhibiting the neurons (Kroeger et al., 2018). Thus, in addition to emphasizing the importance of identifying endogenous firing rates of the targeted neuronal population, these results demonstrate that care should be given to stimulate the neurons at a frequency that matches their endogenous firing rate during the behavioral state of interest.

The interpretation of results from chemogenetic or optogenetic inhibition presents another set of challenges. Inhibition using hM4Di DREADDs is known to have variable efficacy, with incomplete suppression of activity of hM4Di-expressing neurons occurring whether CNO is administered locally or systemically (Mahler et al., 2014; Chang et al., 2015; Cichon and Gan, 2015). Our own hM4Di results reflect this, with the number of c-Fos-expressing POA Tac1 neurons decreasing by only 30% after systemic administration of 3 mg/kg CNO. Incomplete inhibition may not be enough to alter behavior, given that many of the neurons of interest remain active. While optogenetic inhibition with halorhodopsin or archaerhodopsin may be more effective at silencing neuronal activity than chemogenetic inhibition, the effect of photoinhibition is more spatially limited, only to the area illuminated by the laser. Thus, the laser penetration may not be enough to effectively silence the entire POA.

Ultimately, the benefits and drawbacks of each method must be weighed carefully in order to select the most appropriate technique for a given experiment, and comparisons of studies using each technique when investigating POA regulation of arousal state will be important to fully untangle its role.

## Technical Innovations

New innovations in biomedical science and equipment are bringing the field ever closer to untangling the role of the POA in natural and anesthetic-induced arousal state regulation. The use of more temporally specific measurements of neuronal activity, such as calcium imaging and *in vivo* electrode recordings in freely behaving animals, now allows for examinations of POA neuronal activity across sleep stages as well as anesthetic induction, maintenance, and emergence. Given evidence that the neural circuitry involved in anesthetic induction may not be identical to that involved in emergence (Kelz et al., 2008; Dong et al., 2009; Gompf et al., 2009; Friedman et al., 2010; Zhang et al., 2012, 2016), the ability to record neuronal activity across each of these phases will be an invaluable contribution to understanding anesthetic mechanisms. Additionally, advancements in neural circuit mapping such as channel rhodopsin-assisted circuit mapping (Petreanu et al., 2007) will aid in distinguishing functional projections to and from the POA that may help mediate the region’s effects on arousal state.

One exciting area of technical innovation lies in techniques linking immediate early gene expression to an effector molecule, controlled by pharmacological treatment. These have been discussed in detail in a recent review (Franceschini et al., 2020), so we will only highlight a selection here. One technique that has already been utilized to study the POA is the TetTag system (Reijmers et al., 2007). This system uses the *Fos* promoter to drive the expression of a tetracycline transactivator in the absence of doxycycline. Thus, this system is switched off in the presence of doxycycline, which can be administered in a number of ways. Once doxycycline is removed and a c-Fos activating stimulus occurs, the tetracycline transactivator is expressed, ultimately driving the expression of a downstream effector molecule, which can include optogenetic or chemogenetic tools (Reijmers et al., 2007).

This TetTag system has been used to study the relationship between sleep and dexmedetomidine-induced sedation in the POA. Neurons tagged with excitatory DREADDs during dexmedetomidine sedation and later reactivated promote sleep, demonstrating that an identical population of neurons is involved in both states (Zhang Z. et al., 2015). Given that dexmedetomidine produces a type of sedation distinct from other general anesthetics, the degree to which this result can be generalized to other anesthetics is not clear given the distinct types of unconscious induced by each. Still, these results highlight the utility of this technique when investigating arousal state regulation.

Another drug-dependent immediate early gene-linked technique is *targeted recombination in active populations* (TRAP), which shows improved temporal resolution compared to TetTag (Guenthner et al., 2013). This technique places a tamoxifen-inducible recombinase under control of an immediate early gene reporter such as c-Fos. Thus, the recombinase is only active in the presence of tamoxifen, the administration of which is controlled by the experimenter. By coupling this technique with optogenetic and chemogenetic effector molecules, neuronal populations active during a specific task or time can be TRAPed and later reactivated (Franceschini et al., 2020). Additionally, neuronal populations can be TRAPed with a fluorescent molecule during one stimulus and this fluorescent pattern can be compared to a c-Fos signal induced by a later, second type of stimulus. A new version of TRAP (TRAP2) was also recently developed that exhibits enhanced effector expression and improved penetration in many brain regions (DeNardo et al., 2019).

Finally, another recently developed immediate early gene-linked tool has already been used to study sleep and anesthetic mechanisms. This technique, called *capturing activated neuronal ensembles with engineered mice and viruses* (CANE), inserts a destabilized avian tumor virus receptor A (TVA) under the control of the Fos promoter (Sakurai et al., 2016). Thus, the TVA is only expressed in activated neurons, and only for a window of a few hours until the TVA is degraded. A virus pseudotyped with the ligand of TVA, EnvA, is injected into the brain region of interest and infects neurons that express TVA during the injection window. This virus can carry fluorescent proteins to label the active neurons, or chemogenetic or optogenetic effector molecules to enable subsequent activation or inhibition of this tagged population (Sakurai et al., 2016; Jiang-Xie et al., 2019). Though not in the POA, this technique identified a population of anesthetic-activated neurons in the neighboring supraoptic nucleus that promote NREM sleep when reactivated at a later time (Jiang-Xie et al., 2019), highlighting the power of this technique in investigations of the shared circuitry hypothesis in the POA and other regions.

## Conclusion

Understanding the role of the POA in regulating arousal state is a critically important topic, given the range of consequences that result from improper arousal state regulation. Each year a small but nevertheless significant number of patients experience undesirable arousal state transitions in response to general anesthesia. Such patients may regain consciousness during surgery (Sebel et al., 2004; Mashour and Avidan, 2015; Sanders et al., 2017) or exhibit delayed emergence from the anesthetic state (Mesa et al., 2000; Cascella et al., 2018). Thus, understanding the mechanisms by which general anesthetics alter the arousal state of an organism, producing a state of unconsciousness, is an important medical question to ultimately reduce or prevent these inappropriate arousal state transitions from occurring. Additionally, insights into the mechanisms of anesthetic-induced unconsciousness will have important implications for our understanding of the neural basis of consciousness and natural arousal state regulation itself, as well as disorders of consciousness such as coma and sleep disorders. With the continuous improvement and development of technical methods and an improved ability to distinguish and target arousal state-regulating neurons, our understanding of the exact role that POA plays in regulating arousal states under natural sleep and wake as well as general anesthesia is closer than ever.

## Author Contributions

SR contributed to the conceptualization of the manuscript, wrote the initial draft, contributed to editing, and prepared the figures. MK contributed to the conceptualization, reviewed, and edited the manuscript. Both authors approved the final manuscript.

## Conflict of Interest

The authors declare that the research was conducted in the absence of any commercial or financial relationships that could be construed as a potential conflict of interest.
